# Reconstruction of a subtotal upper lip defect with a facial artery musculomucosal flap, kite flap, and radial forearm free flap: a case report

**DOI:** 10.1186/s12957-018-1492-5

**Published:** 2018-09-28

**Authors:** Shuai Wang, Zeliang Zhang, Zhongfei Xu, Weiyi Duan

**Affiliations:** 1 Department of Oromaxillofacial-Head and Neck Surgery, No. 117, Nanjing North Street, Heping District, Shenyang, Liaoning 110002 People’s Republic of China; 2 Department of Oral Maxillofacial Surgery School of Stomatology, China Medical University, No. 117, Nanjing North Street, Heping District, Shenyang, Liaoning 110002 People’s Republic of China

**Keywords:** Lip reconstruction, Facial artery musculomucosal flap, Kite flap, Radial forearm free flap

## Abstract

**Background:**

For reconstructive surgeons, massive midface defects, including large, full-thickness wounds on the upper lip, can be very challenging. Although there are many methods for reconstruction of upper lip defects, it is difficult to obtain satisfactory restoration of oral functions and good cosmetic results.

**Case presentation:**

This case report presents a man with massive midface defects, including upper lip, left nose, and cheek defects. Over the previous 2 years, the patient had three reconstructions with sequential free flaps for the resection of recurrent tumors, the first of which was in March of 2016; this resulted in the patient having massive midface defects, including an upper lip defect, a defect on the left side of the nose, and one on the left cheek. The defects were reconstructed using a radial forearm free flap (RFFF), a facial artery musculomucosal (FAMM) flap, and a kite flap. In June 2016, he underwent a second reconstruction, this time of the left nose defect, using a left anterolateral thigh (ALT) flap. In March of 2017, the patient underwent a third reconstruction with the use of a free ALT on the left intraoral cheek and the defects on the neck. All flaps survived. No complications were encountered postoperatively. The patient regained good oral sphincter function with no reports of drooling. Although the patient underwent three surgeries, the reconstruction results were acceptable.

**Conclusions:**

For massive midface defects, including large, full-thickness wounds on the upper lip, the combination of a FAMM flap, kite flap, and RFFF promotes the reconstruction of the complex midface structure and improves the resulting functionality.

## Background

The massive destruction that is associated with infections, tumors, trauma, and thermal injuries, even surgical resection itself, results in significantly disfigured three-dimensional (3D) defects that are challenging during reconstruction due to the amount of tissues involved, including the cheek, the nose, and upper lip, all of which require reconstruction [1]. The intentions and the principles of a midface reconstruction are to achieve adequate functioning along with esthetics. All the procedures involved make reconstruction of either or both the upper and bottom lips, and an achievement of good functional and esthetic results is a daunting task. The function of the oral sphincter includes sensation, movement, color, and appearance [2]. There are various methods available, including local, regional, and microvascular free flaps, to achieve reconstruction for defects of the lip, as well as for midface defects. The local and regional flaps do not usually provide an adequate amount of support, coverage, or lining for large defected areas, even though there would be good color and texture matching. The conventional free flaps are not sufficient to replace the original multilayered facial structures [2]. Dr. Corderio and Dr. Jeng reported a method called the “combination method,” involving a local flap for lip reconstruction and a free flap for patients presenting with massive midface or lower face defects [3–5]. For massive midface defects, including the somewhat larger areas with defects having a full thickness on the upper lip, the use of a combination of the FAMM flap, kite flap, and the RFFF appears to be a beneficial method.

## Case presentation

**Fig. 1:**
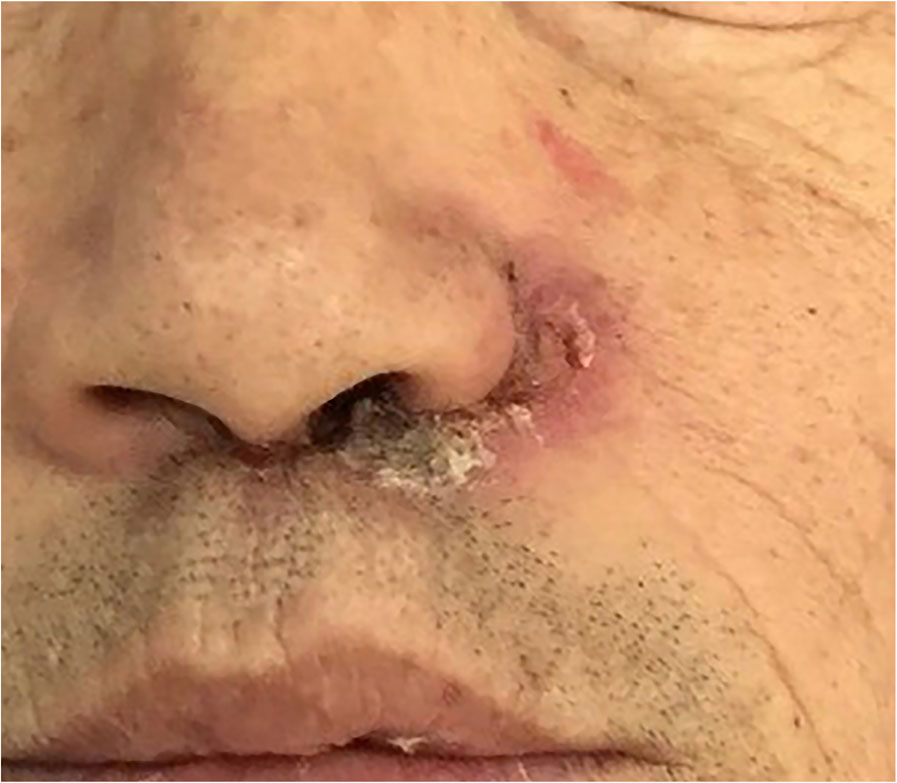
A 61-year-old man presented with squamous cell carcinoma in the left nose region. The tumor was ulcerated through the skin of the left nostril and upper lip

**Fig. 2:**
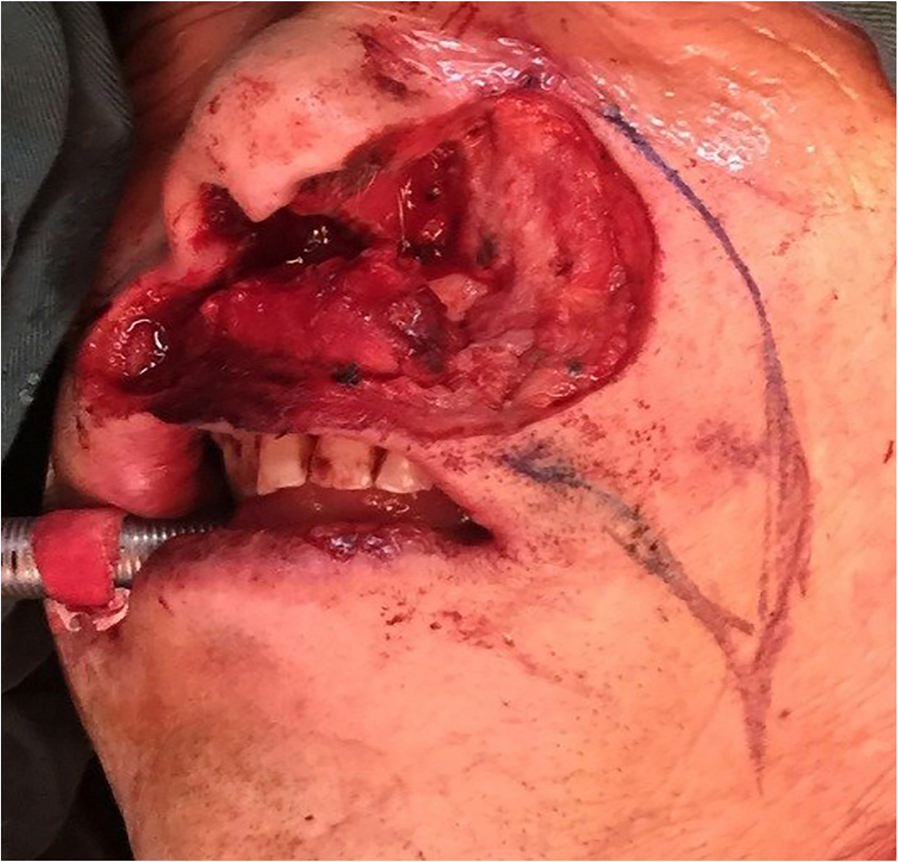
Tumor ablation: the defects included the upper lip, left nose, and left cheek

The subject was a man with a mass in his left nostril for at least 1 year. He came to our hospital for treatment in March 2016 when he was 61 years old. He reported he had been experiencing pain and itching that he could not relieve with medications. The mass was a tumor that had ulcerated through the skin in the last half year (Fig. 1). He underwent a preoperative biopsy at another medical institution, and the result showed squamous cell carcinoma (SCC) in the left nose region. Upon palpation of the left side of his neck, the examining doctor felt lymph nodes located in the left submaxillary region. When performing a preoperative evaluation, the patient was diagnosed as having an SCC in the left nose region, with the tumor being stage T2N1MO. Once the ablation was completed, a very large soft tissue defect remained that spanned the upper lip, the left side of the nose, and the left cheek. The defect of the upper lip occupied approximately 60% of the area (Fig. 2).

**Fig. 3:**
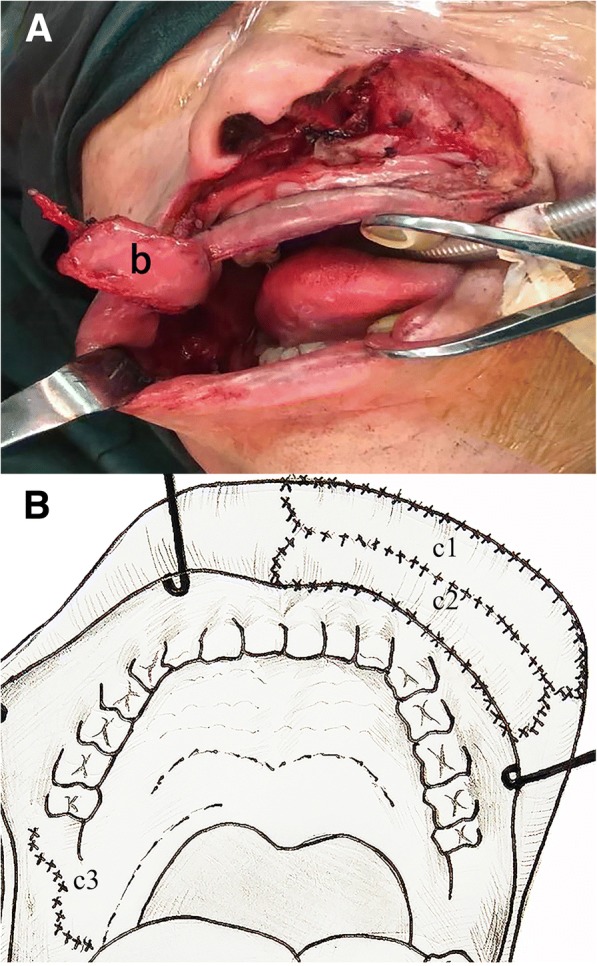
**a** The FAMM flap: the flap (**b**) was harvested from the right intraoral cheek and measured 2 × 5 cm^2^. **b** Reconstruction of the vermilion line, labial sulcus, and the oral sphincter: c1, the remainder mucosa of the vermilion; c2, a FAMM flap; and c3, the donor site of the right cheek was closed primarily

To reconstruct these defects, we had to divide the procedures for the reconstruction into two parts. The first part of the reconstruction procedures was to reconstruct the upper lip defect. To restore the vermilion line, labial sulcus, and oral sphincter, we mobilized the remaining mucosa of the vermilion, keeping its blood supply intact through its lateral pedicles, and this part was used to restore the vermilion lip border. The facial artery was located preoperatively by Doppler ultrasound, helping to ensure that the artery remained in the FAMM flap throughout the harvest. We designed a 2 cm × 5 cm flap on the cheek mucosa. The flap axis crossed the projection of the facial vessels at approximately 90°. To obtain both static and dynamic tonus of the flap, the buccinator of the FAMM flap was sutured to the upper orbicularis muscles in each remaining site with 4–0 polydioxanone bilaterally. The mucosa, belonging to the FAMM flap, was sutured to the residual intraoral mucous to restore the defects of the vermilion and the upper lip mucosal lining. The right cheek donor site was primarily closed (Fig. 3).

**Fig. 4:**
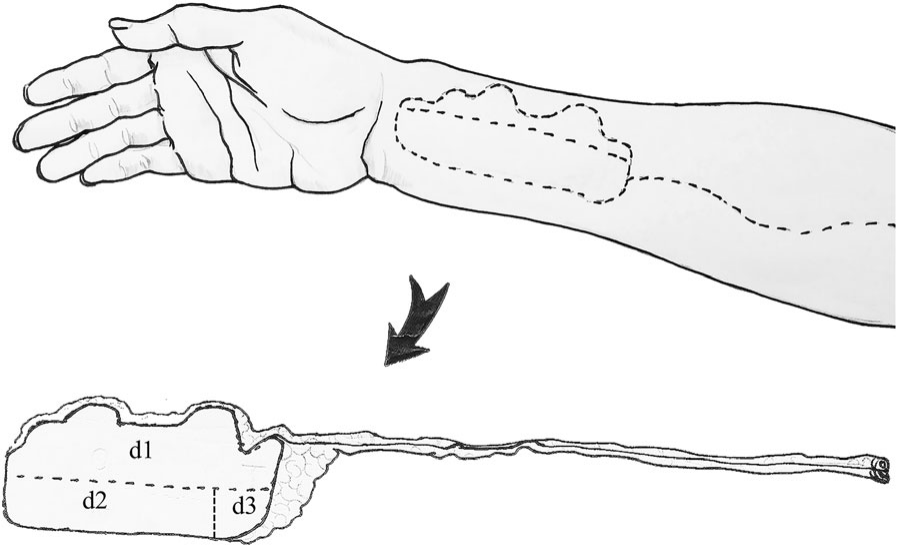
A RFFF from the right forearm was designed according to the size and shape of the central face defect

**Fig. 5:**
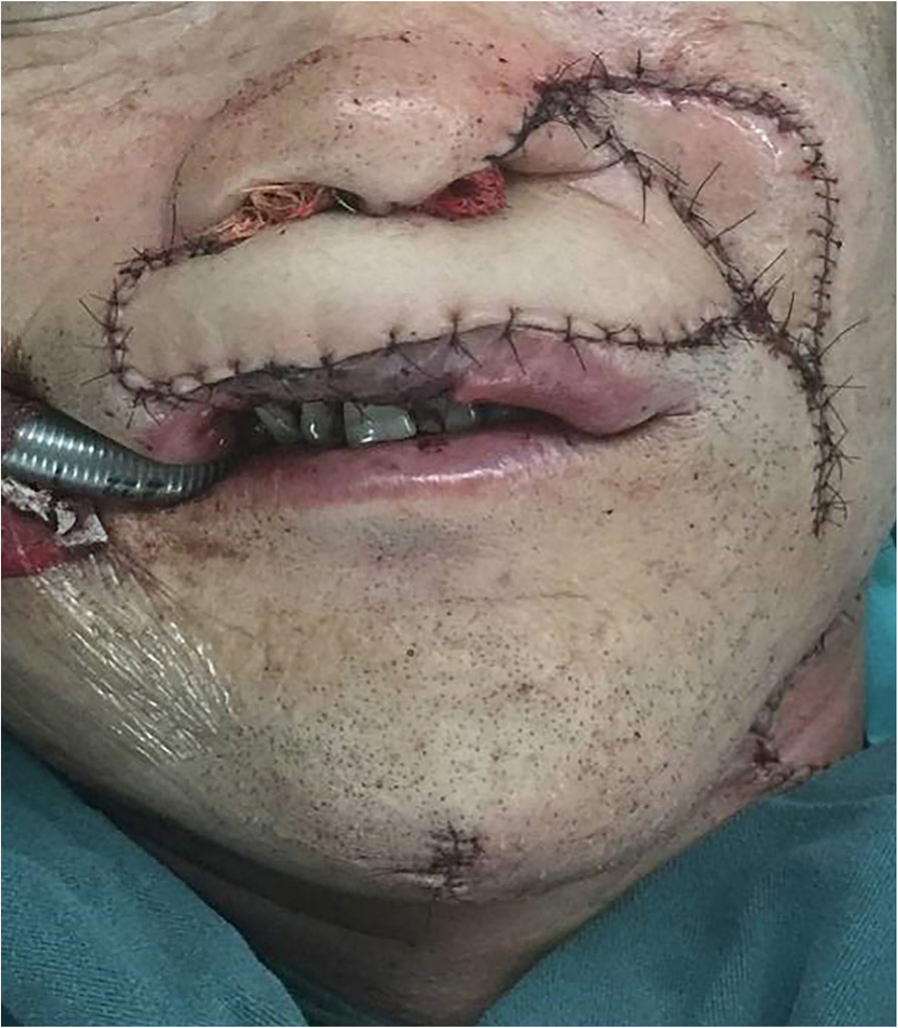
External effect after reconstruction

The second procedure for the reconstruction involved reconstruction of the cheek defects, the defect of the left nostril in the nose, and for the defect in the external skin on the upper lip. According to the shape and size of the midface defect, we designed a RFFF (Fig. 4). The size of the RFFF was 40 cm^2^ (5 × 8 cm). We were able to revascularize by the end-to-end microvascular anastomoses of the lingual artery and a branch belonging to the internal jugular vein, with the cephalic vein being anastomosed together with the external jugular vein. The flap was then divided into three sections. The folding section (d1) was used in the reconstruction of the nasal floor and the nasal ala. Section d2, was used in the reconstruction of the external skin on the upper lip, and the inferior border of the RFFF was sutured together with the upper border for the remaining mucosa belonging to the vermilion. A kite flap based on the left facial artery was designed and harvested along the left nasolabial fold. The kite flap was sutured with portion d3 of the RFFF to repair the cheek defect and to constitute the lower alar contour after advancement. After reconstruction, two plastic tubes were placed on either side of the nostrils to facilitate the formation of the nasal shape and to help the patient breathe (Fig. 5). When the initial reconstruction was over, the report from the pTNM confirmed that his tumor was a moderately differentiated SCC in the extraoral cheek, left nostril, and the upper lip and was graded as T2N1M0. The patient underwent an R0 resection.

**Fig. 6:**
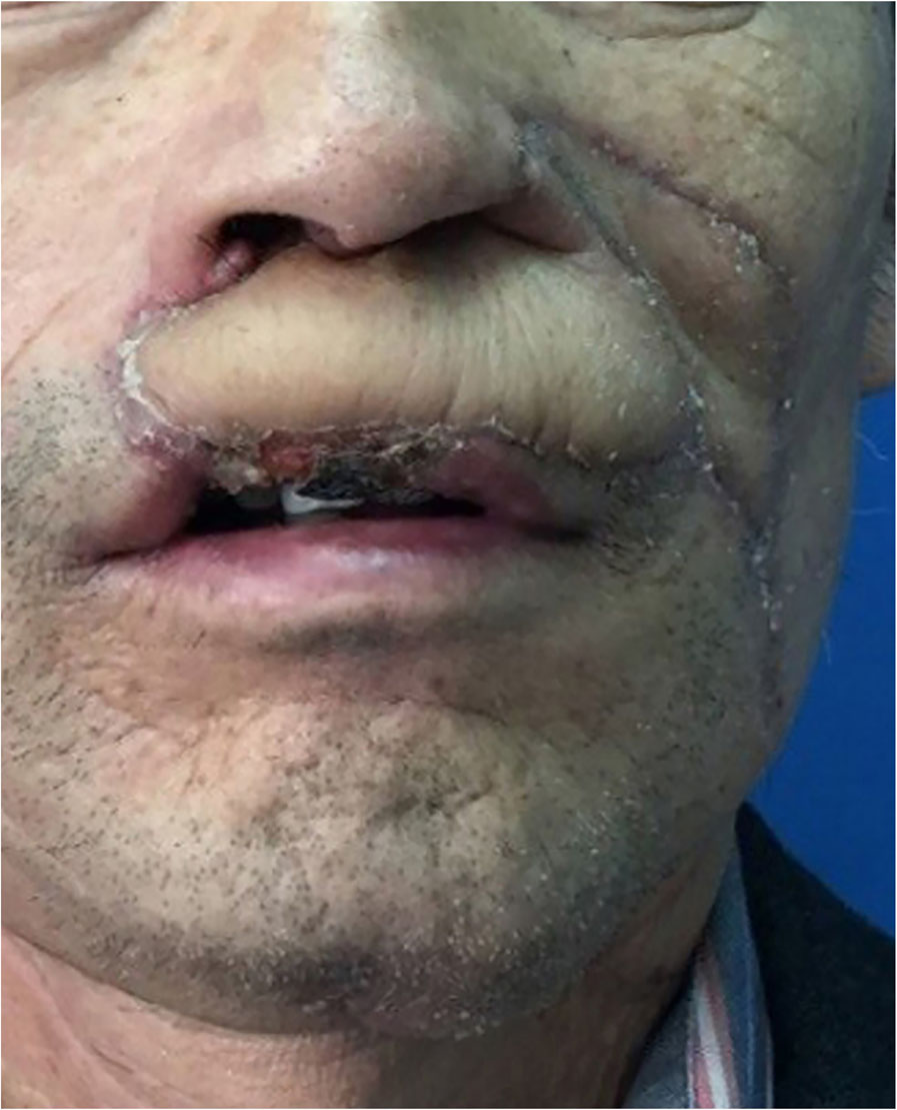
Fifteen days after the first operation

There were no complications postoperatively. All flaps extraordinarily survived. The patient started on a liquid diet once he awoke from the anesthesia. On the 9th postoperative day, soft foods were administered, and the patient was discharged shortly thereafter. The stitches in the forearm and abdomen were removed on postoperative day 15 when he returned to our hospital for a check-up (Fig. 6). Oral functions, including grinning, pouting, and protrusion, as well as cosmetic appearance, including restorative results of the vermillion, were satisfactory. Mouth opening was normal at 3.5 cm. This patient gained good oral sphincter functions, with no drooling reported.

**Fig. 7:**
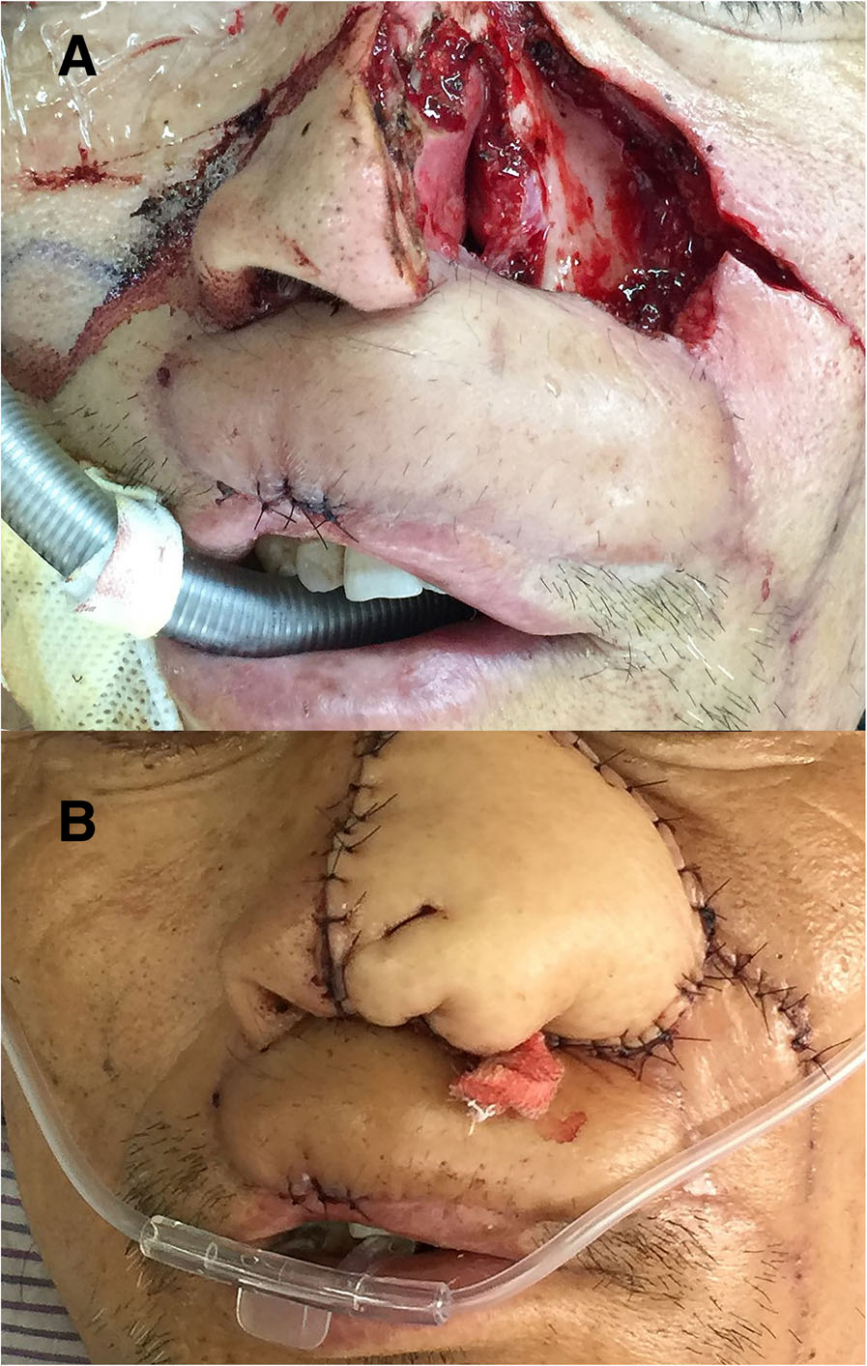
**a**, **b** The patient underwent his second reconstruction of the left nose defect by using a thinned anterolateral thigh

The patient refused to receive radiotherapy after the reconstruction surgery. Unfortunately, he was diagnosed with a recurrent squamous cell carcinoma in the left nostril only 3 months after the first reconstruction operation and was hospitalized a second time on June 6, 2016. The resection of this tumor resulted in a defect that involved the external skin covering the left cheek and the left nostril. The patient also underwent a second reconstruction on the left nose defect, performed using a thinned anterolateral thigh (ALTF) taken from the left thigh (Fig. 7). It was anastomosed to his left superficial temporal veins and arteries. Although two plastic tubes were placed on both sides of the nostril to facilitate the formation of the nasal shape and help the patient breathe after the reconstructive operation, the left nostril appeared narrow.

**Fig. 8:**
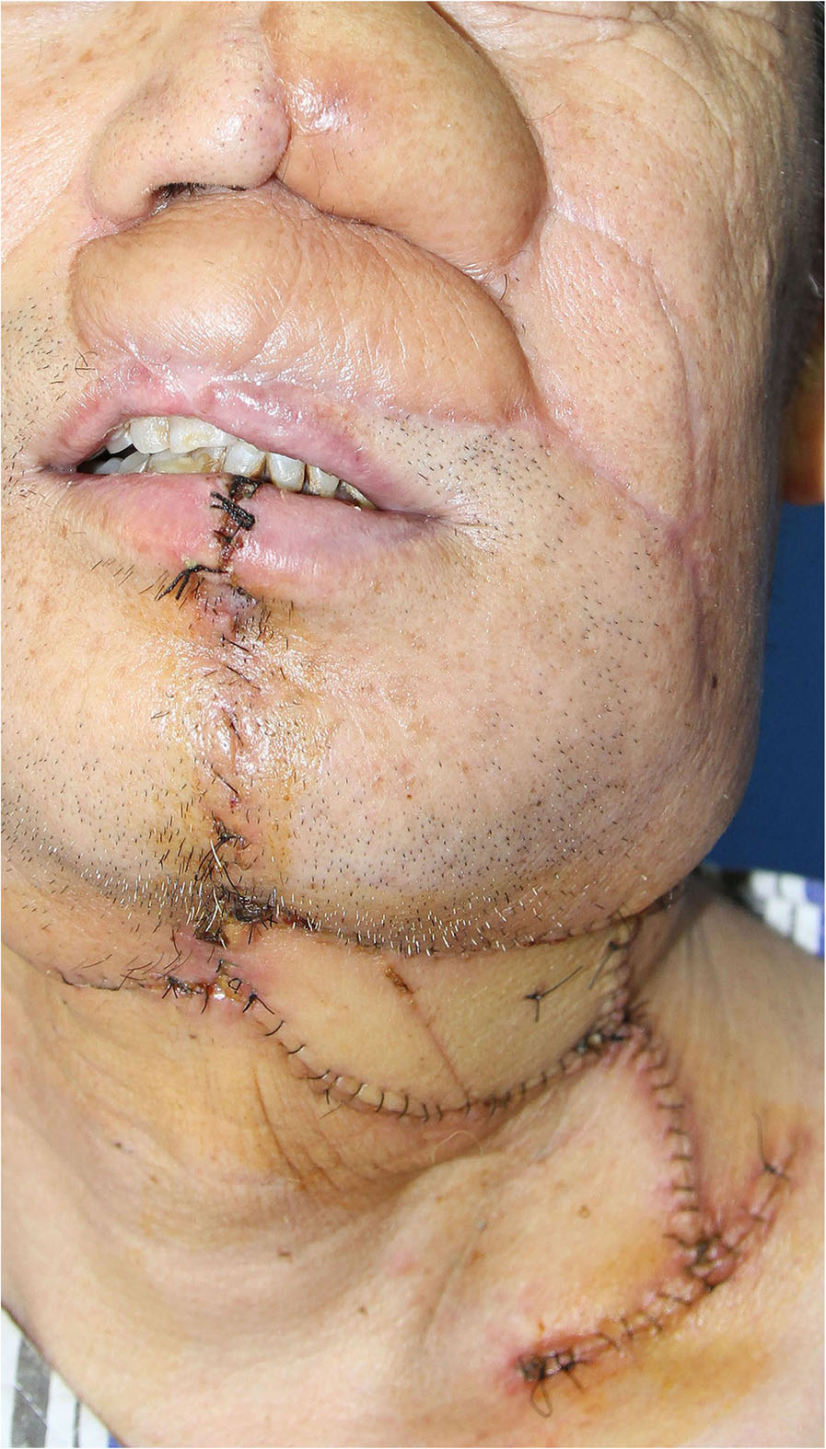
The patient underwent a third reconstruction with free right ALT for the left intraoral cheek and neck defects. One-week postoperative photograph of the patient

After the second reconstruction, the patient once again refused radiotherapy, and his cancer reappeared after 9 months of remission. The patient underwent a third reconstruction using a free right ALT as the left intraoral cheek and for the neck defects in March of 2017. The ALT flap carried two skin paddles, each of which had an independent perforator [6]. This flap was tunneled to the transverse cervical artery and vein (Fig. 8).

**Fig. 9:**
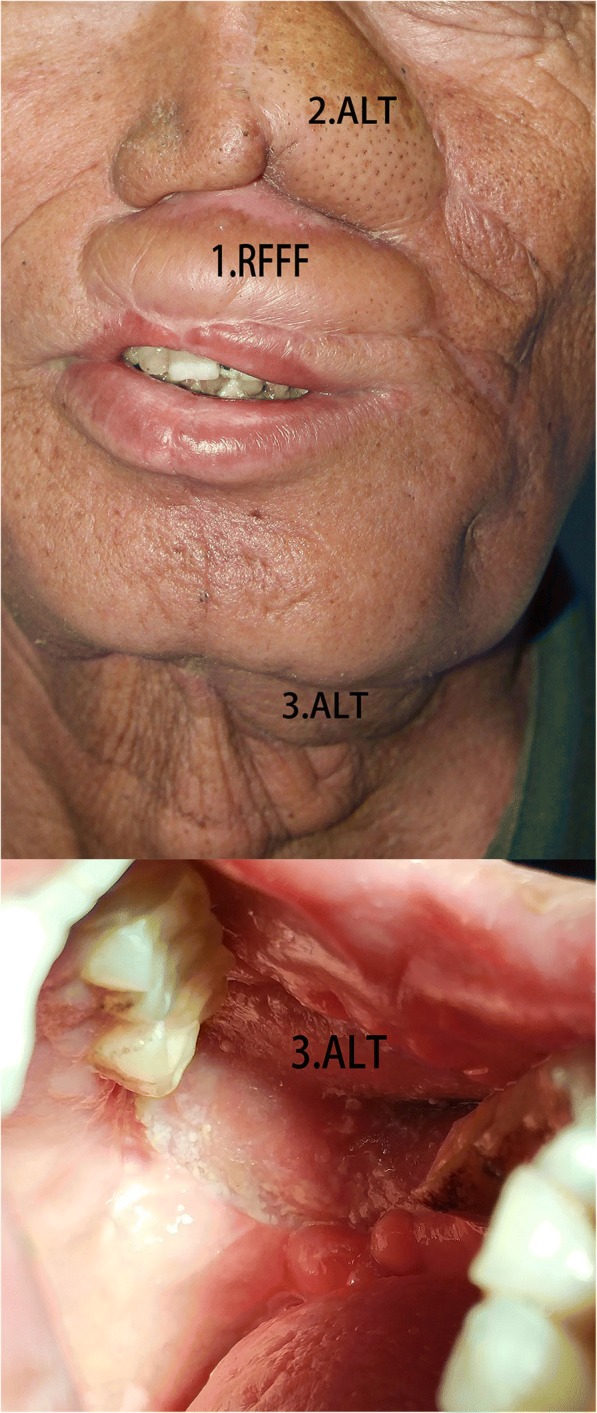
One month after the radiotherapy. Numbers 1–3 represent the sequence and types of flaps used in the reconstruction

After undergoing his third reconstruction, the patient received the radiotherapy. However, only 1 month later, due to a flap retraction, the patient had incomplete closure of his mouth, but there was no report of drooling. The patient was satisfied with the results of the reconstruction, particularly with the esthetic effect (Fig. 9).

## Discussion and conclusions

Massive midface defects that involve the lip(s) can pose challenging reconstructive problems. This region’s visibility makes the cosmetic outcome of its reconstructions more important than those of any other region of the body. Free tissue transplantation is the preferred method to reconstruct massive dentofacial defects, and local tissue should be used wherever possible. The lips are considered the central feature of the face. It is difficult to reconstruct lip defects as they are complex structures composed of muscle, fat, vermilion, mucosa, and skin. When performing reconstruction, it is best to use identical or similar tissues. When a plastic surgeon attempts to perform reconstruction of massive midface defects, including large upper lip defects, it is necessary to make careful preoperative plans in order to restore functions and appearances. Generally, for lip reconstruction, defects involving less than a third of the lip can be closed primarily. If the defect is large (30 to 80%), Estlander, Abbe, or Karapandzic flaps are used [6]. Moreover, if the surgeon decides to reconstruct larger lip defects with local flaps, he or she must consider the disadvantages of rotating large amounts of local tissue, including facial disfigurement, microstomia, and often unfavorable results.

For this case report, the surgeon chose the FAMM flap for restoring the vermilion line, mucosal lining, and oral sphincter. Pribaz et al. were the first to provide details regarding the FAMM flap in 1992. New modifications were developed with subsequent publications, and the FAMM flap became more versatile for use as an intraoral musculomucosal flap [7, 8]. Tareck et al. reported a versatile reconstruction option called the FAMM flap for use in small- and medium-sized defects in the oral cavity, lips, posterior skull base, oropharynx, nasal septum, and many other sites that are less commonly exploited [8–12].

Many studies reported the use of several local flaps to reconstruct midface defects. Daniel et al. reported that the nasolabial flap combined with the FAMM flap can be applied to perioral and intraoral defects [13]. However, its disadvantages are also apparent: it cannot be used to restore massive defects of the midface. Methods for oral reconstruction by improving free flaps have been reported. Chien and Onder used the ALT flap for the reconstruction of lip defects [14, 15]. Reconstruction of midface defects through RFFF was described by Wei [2]. However, these procedures only provide lip anatomical morphology without the dynamic function that is necessary for normal oral sphincter function. Nevertheless, the free gracilis muscle flap, when combined with the forearm flap, has been shown to be successful in reconstruction, and the reconstructive method modifications have been limited [16–19]. Moreover, whether using RFFF, ALT, or any other free flaps, when used to reconstruct the upper or lower lip, it is difficult to restore the texture of the mucosa.

More recently, free flap and local flap transfers have been used for extensive lip and perioral defects reconstruction, yielding superior results [3–5]. Attempts at reconstructing complex defects with free flaps and lip-switch procedures have also provided superior outcomes [3, 20]. Nevertheless, both the lower and upper lips of a typical lip-switch flap repair result in smaller postoperative horizontal breadths. Therefore, significant microstomia functions could occur if a broad lip wound was repaired using a lip-switch flap. Milomir and Adriana used the FAMM flap combined with the gracilis muscle flap to reconstruct large defects of the lower lip, and they achieved an ideal effect [16, 21]. There are no reports regarding the reconstruction of massive midface defects, including upper lip defects, by combining the FAMM flap with a free flap. Therefore, we used the FAMM flap, the kite flap, and the RFFF to reconstruct the current patient’s lip defect and massive midface defects on March 16, 2016.

In this case, the patient had very high expectations for the esthetic results of the reconstruction. The functional and esthetic results achieved for the patient originated from meticulous preoperative planning. The RFFF is primarily used for head and neck reconstruction because of its large size, thin and flexible tissue, abundant blood supply, and long vascular pedicle. Based on the shape and size of the patient’s central face defect, the RFFF of the right forearm was designed [22]. The RFFF was folded to restore the shape of the nose, while the kite flap was harvested and sutured to the border of the free flap [23–26].

Forming the oral sphincter and vermilion lip border is difficult. In this situation, we repaired the external skin of the upper lip by the free flap and restored the orbicularis oris muscle and the inner lining of the mucosa using the FAMM flap. Finally, we mobilized the remaining mucosa of the vermilion and advanced it to restore the vermilion lip border. A satisfactory postoperative result was obtained when we reconstructed the lip and midface defects using two local flaps and a partially folded free flap. The patient obtained a functioning postoperative oral sphincter with no drooling. With the surgery on the vermilion lip border completed, the patient had an ideal appearance to the lips.

Excisional facial operations with loss of defining facial features can leave patients severely disfigured and insecure because of their appearance. When considering a patient’s expectations regarding appearance and when discussing the use of these reconstruction techniques, acknowledging the patient’s goals and expectations of the final results is important [27]. Therefore, in the present case, it was necessary to have good doctor-patient communications because the patient underwent the second and third surgical reconstructions and resections.

The FAMM flap, kite flap, and RFFF were chosen for reconstruction of the upper lip and midface defects. These three flaps offer advantages that outweigh the disadvantages. Furthermore, they offer predictable anatomy with good arterial supply. In addition, morbidity occurring in the donor site is rare, and the surgeons had experience with the harvested flaps. Therefore, the advantages resulted in an excellent success rate using the flaps for reconstruction. For an experienced head and neck reconstructive surgeon, whether the nasolabial flap, FAMM flap, or radial forearm free flap is used, it is a “see one, do one, teach one” type of procedure that should be a skill for any surgeon in the field. When the flaps are combined by a plastic surgeon for restoring head and neck defects, the technical difficulty is not much higher than using any one of them individually and can be considered similar to “a jigsaw puzzle.” Nevertheless, adequate preoperative evaluations and planning of reconstructive surgical procedures are important. In this case report, we introduced a reliable and effective method to handle reconstruction cases that involved extensive midface resection including the upper lip.

Overall, in cases involving massive midface resection, including resections of the upper lip, this method of reconstruction combining the FAMM flap, the kite flap, and the radial forearm free flap can be useful.

## Abbreviations

3D: 3-Dimensional
ALT: Left anterolateral thigh flap
FAMM: Facial artery musculomucosal flap
RFFF: Radial forearm free flap
SCC: Squamous cell carcinoma
